# Redundant sensory information does not enhance sequence learning in the serial reaction time task

**DOI:** 10.2478/v10053-008-0108-y

**Published:** 2012-03-01

**Authors:** Elger L. Abrahamse, Rob H. J. van der Lubbe, Willem B. Verwey, Izabela Szumska, Piotr Jaśkowski

**Affiliations:** 1 Department of Experimental Psychology, University of Ghent, Ghent, Belgium; 2 Department of Cognitive Psychology and Ergonomics, University of Twente, Enschede, The Netherlands; 3 Department of Cognitive Psychology, University of Finance and Management, Warsaw, Poland

**Keywords:** sequence learning, implicit learning, sensory redundancy, serial reaction time task

## Abstract

In daily life we encounter multiple sources of sensory information at any given moment. Unknown is whether such sensory redundancy in some way affects implicit learning of a sequence of events. In the current paper we explored this issue in a serial reaction time task. Our results indicate that redundant sensory information does not enhance sequence learning when all sensory information is presented at the same location (responding to the position and/or color of the stimuli; Experiment 1), even when the distinct sensory sources provide more or less similar baseline response latencies (responding to the shape and/or color of the stimuli; Experiment 2). These findings support the claim that sequence learning does not (necessarily) benefit from sensory redundancy. Moreover, transfer was observed between various sets of stimuli, indicating that learning was predominantly response-based.

## INTRODUCTION

*Implicit learning* refers to the phenomenon that people are able to acquire skilled behavior or structured knowledge about their environment in a seemingly automatic and unconscious fashion. Over the last decades, the serial reaction time (SRT) task has become a highly productive tool in the investigation of implicit learning (e.g., Nissen & Bullemer, 1987; for overviews, see Abrahamse, Jiménez, Verwey, & Clegg, 2010; Clegg, DiGirolamo, & Keele, 1998; Keele, Ivry, Mayr, Hazeltine, & Heuer, 2003). In this task, participants are required to respond fast and accurately to a particular feature of successively presented stimuli on a screen - here referred to as *response cues*^1^. Unknown to the participants, the order of presentation of these response cues is pre-structured, thereby allowing learning of the structure across training (i.e., *sequence learning*). To differentiate sequence learning from general practice effects, a block of (pseudo-) randomly selected response cues is inserted near the end of the practice phase. The cost in reaction time (RT) and/or accuracy of this random block relative to its surrounding sequence blocks is commonly used as an index for sequence learning. Notably, sequence learning (as indicated by performance measures) and sequence awareness (i.e., explicit knowledge about the precise regularity) often do not develop at the same rate during training: Relatively small increases in awareness are accompanied by substantial increases in response speed and accuracy. Learning is therefore said to be (partly) implicit.

In recent years several authors tried to place the SRT task into an ecologically more valid context by moving away from the typical simple key-presses in response to simple, single response cues on the screen. For example, implicit sequence learning has been observed in SRT settings that involved more complex and/or continuous actions than discrete key-presses (e.g., Chambaron, Ginhac, Ferrel-Chapus, & Perruchet, 2006; Witt & Willingham, 2006). In addition, from the notion that in real life we mostly move around in a perceptually rich environment, Jiménez and Vázquez (2008) combined the SRT task with a visual search paradigm. They observed that the presence of distracter elements on the screen did not hinder implicit sequence learning. In the current paper we further pursued the issue of sensory information by exploring how multiple temporally synchronized and congruent response cues - further referred to as *redundant response cues* - may combine to affect implicit sequence learning. We believe that this, first, further extends the issue of ecological validity, and second, contributes to a large literature on the role of sensory information in implicit sequence learning (e.g., Clegg, 2005; Deroost & Soetens, 2006a, 2006b; Remillard, 2003).

For years it has been debated whether implicit sequence learning (in the SRT task) is mainly stimulus- or response-based. Even though response-based sequence learning (e.g., learning a sequence of successive response locations) is typically viewed as the dominant form of learning in the SRT task since a set of studies by Willingham and colleagues (Bischoff-Grethe, Goedert, Willingham, & Grafton, 2004; Willingham, 1999; Willingham, Wells, Farrell, & Stemwedel, 2000), support for a significant role of sensory information in sequence learning is now so strong that it should not be ignored (e.g., Clegg, 2005; Deroost & Soetens, 2006b; Nemeth, Hallgató, Janacsek, Sándor, & Londe, 2009; Remillard, 2003; Song, Howard, & Howard, 2008; Ziessler & Nattkemper, 2001). Moreover, the major model on sequence learning to date - the dual system model by Keele et al. (2003) - considers both stimulus- and response-based processes. Specifically, in this model a (non-specified) number of separate unidimensional modules are presumed to detect and utilize all available regularity within particular types of stimulus- or response-based information, whereas a multidimensional module additionally allows sequence learning across types of information. This strongly relates to the current study, as it actually predicts that redundant response cues could - in theory - produce better sequence learning than single response cues because multiple (instead of one) sensory-specific modules are engaged.

In an attempt to explore this issue empirically, Abrahamse, Van der Lubbe, and Verwey (2009) studied the effect of adding congruent tactile response cues (presented directly to the fingers by using vibrotactile stimulators; cf. Abrahamse, Van der Lubbe, & Verwey, 2008) to the (visual) position response cues of an otherwise standard SRT task. It was observed that this addition did not affect the amount of sequence learning as compared to conditions in which either only visual or only tactile cues were employed. This could be a first indication that sequence learning in an SRT task does not typically benefit from multiple congruent response cues, and thus that implicit sequence learning is not so unselective after all. However, as noted already by Abrahamse et al. (2009), some alternative explanations may be considered. First, sequence learning benefits may have been absent in our previous study because of the spatial disparity between the employed response cues: Position response cues were presented on the screen, while tactile response cues were presented to the fingers. This spatial disparity may have (a) rendered integration of both response cues difficult, as spatial correspondence is thought to be an important determinant of sensory integration (e.g., Driver & Spence, 2000; Radeau, 1994; Stein & Stanford, 2008), and (b) forced participants to restrict themselves to one modality as it may be hard to divide attention across two locations (Posner, 1980). Experiment 1 of the current study addressed this issue by employing redundant response cues that are always presented at the same location. As an alternative explanation, it may be that sequence learning effects were obtained independently for both the visual and tactile response cues, but that a redundancy gain was not observed because baseline response latencies of one type of response cue (i.e., the tactile response cue) were too large to (substantially) contribute to general performance. This second possibility will be addressed in Experiment 2.

## EXPERIMENT 1

The aim of Experiment 1 was to explore the effect of congruent, spatial-temporally coinciding response cues on sequence learning in the SRT task. This was achieved by using position and color cues; that is, each response was mapped exclusively onto a stimulus with a specific color that appears at a specific position, so that the correct response is simultaneously signaled both through the position and the color of the stimulus. This design has been employed already in a set of studies by Robertson and colleagues (Robertson & Pascual-Leone, 2001; Robertson, Tormos, Maeda, & Pascual-Leone, 2001), who reported better sequence learning in the combined position and color cue condition than in either of the single cue conditions. However, the conclusiveness of their findings is unclear after a detailed look at these studies.

First, Robertson et al. (2001) employed probably more difficult sequences (e.g., “4-1-2-4-3-2-1-4-1-3”) in their single response cue conditions than in their combined position/color response cue condition (e.g., “2-1-3- 2-4-3-1-3-2-4”). As a consequence, the observation of more pronounced sequence learning in the latter condition may be attributable to the relatively easy sequences used in that condition. Second, Robertson and Pascual-Leone (2001) chose to analyze Z-transformed scores instead of absolute differences in RT in order to normalize differences in baseline response latencies (please note that the sequence learning effect in absolute RTs amounted to 176 ms for participants training with combined color and position cues, and to 186 ms for participants with only color cues.). This transformation would be justified from the assumption that sequence learning is better expressed in a task with a larger baseline RT than in a task with a smaller baseline RT: Taking baseline RT into account by performing normalization, then, would compensate for these assumed differences in the expression of sequence learning. To the best of our knowledge, however, there is no direct empirical support in the literature to justify this assumption. Therefore it remains difficult to determine how to best compare sequence learning between group of participants with large differences in baseline response latencies. Additionally, baseline response latencies in the SRT task are characterized by substantial individual differences, even when performing the precise same task. By taking into account accidental differences in baseline response latencies in studies employing a small number of participants (i.e., four participants in each between-subject condition for the study of Robertson & Pascual-Leone, 2001; and six participants in a within-subject design for the study of Robertson et al., 2001), then, the sequence learning effect might have been artificially in- or deflated, rendering an interpretation of results to be difficult.

In Experiment 1 of the current study, we further explored the use of congruent position and color cues. As in the study of Robertson and Pascual-Leone (2001), participants were trained in an SRT task either while responding to position cues (position training group), to color cues (color training group), or to a combination of these cues (combined training group). Hence, in the latter training group, position and color cues were perfectly correlated. However, in contrast to the study by Robertson and Pascual-Leone, after the training phase all participants were tested in all three response cue conditions (the order counterbalanced across participants) in a transfer phase: a position transfer test, a color transfer test, and a combined transfer test (see the Method section for more detail). Hence, it included a test of transfer to the initial training cue condition, thereby providing a clean baseline condition for transfer. Overall, this transfer phase allowed us to compare performances between training groups when tested on identical tasks with - most importantly - similar baseline response latencies (cf. Abrahamse et al., 2009), thereby circumventing the problem of how to deal with potential differences in baseline response latencies during the training phase (i.e., the choice of analyzing either absolute or standardized scores). One may argue that transferring to a single identical cue condition would already be sufficient to solve baseline problems. However, such a design would not recognize possible interactions between a particular training condition and a particular transfer condition (e.g., Abrahamse et al., 2009) that could confound the results.

A second adjustment compared to Robertson and Pascual-Leone (2001) concerned the choice for a second-order-conditional (SOC) in order to enable the use of the process dissociation procedure (PDP) task (Destrebecqz & Cleeremans, 2001; see below for a detailed description) to assess participants´ awareness of the practiced sequence. The PDP task arguably is a more sensitive test for dissociating implicit from explicit knowledge than, for example, free recall and recognition tasks (see Destrebecqz & Cleeremans, 2001). Though it always remains a tricky issue, measuring awareness is important for current purposes as we mainly aim to explore implicit sequence learning.

In sum, in Experiment 1 the effect of redundant position and color response cues (i.e., redundant sensory information) on sequence learning in the SRT task was explored. If the results from Abrahamse et al. (2009) were indicative of a general absence of learning benefits by using redundant response cues, then we would expect to find no such benefits in Experiment 1, too. However, if the spatial disparity of visual and tactile cues in the study of Abrahamse et al. (2009) - which could have caused problems with the integration of information and/or with attentional selection - was crucial with respect to the absence of potential learning benefits by sensory redundancy, Experiment 1 should allow these learning benefits to emerge.

### Method

#### Participants

Fifty-three undergraduates (42 women, 11 men; *M*_age_ = 24, *SD* = 3.1; three left-handed) from the University of Finance and Management (Warsaw, Poland) gave their informed consent to participate in the experiment in exchange for course credits. They had normal or corrected to normal visual acuity, scored perfectly on a subset of the Ishihara color blindness test (Ishihara, 1993), and were naďve as to the purpose of the study.

#### Stimuli and apparatus

Stimulus presentation, timing, and data collection were achieved using the Presentation 10.1 experimental software package on a stan-dard Pentium© IV class PC. Stimuli were presented on a 22 inch Mitsubishi Diamond Pro 2070SB display running at 1,024 by 768 pixel resolution in 32 bit color, with a refresh rate of 120 Hz. Viewing distance was approximately 60 cm (not strictly controlled). Depending on the specific experimental group, placeholders consisted either of (a) a horizontally outlined array containing four grey-lined squares (3 × 3 cm) filled with white, continuously presented on a black background, or (b) a single grey-lined square (3 × 3 cm) filled with white presented on a black background. Stimuli consisted of the appearance in one of the square placeholders of a circle (with 2.5 cm diameter) that was colored purple, red, blue, green, or yellow, depending on the specific experimental group.

#### Procedure

Participants were randomly assigned to one of three experimental groups for the training phase, in which an SRT task was performed: the position training group (*n* = 18), the color training group (*n* = 16), or the combined training group (*n* = 19). In the position training group, participants were instructed to respond to the position of a purple colored circle appearing at one of the four positions of an array, with positions from left to right corresponding to the [*c*], [*v*], [*b*], and [*n*] keys (standard QWERTY keyboard), respectively. In the color training group, participants were instructed to respond to the color of a circle presented in a centrally located square, with colors red, blue, yellow, and green related to the [*c*], [*v*], [*b*], and [*n*] keys, respectively. In the combined training group, each of the four colored (i.e., red, blue, green and yellow) circles was uniquely presented at one of the four array positions, so that participants could employ either the information provided by the position or the color of the circle, or both. The circle remained visible on the screen until responding with a maximum latency of 1,500 ms. After that, the next stimulus would appear with a response-to-stimulus-interval (RSI) of 400 ms. Erroneous responses were signaled to the participants by displaying the word le (Polish word for “error”) right above the placeholders for 1,500 ms, after which the next stimulus was presented at a 1-s interval. Participants responded with the index, middle, ring, and little fingers of their dominant hand.

During the training phase participants performed 10 blocks of 108 trials each. Blocks 1 and 9 were always pseudo-randomly structured; that is, they consisted of a series of nine randomly selected different 12-element SOC sequences, with no element and sequence repetitions allowed. Pseudo-random blocks were never repeated for the same participant. In Blocks 2-8 and Block 10 a 12-element SOC sequence (“242134123143”; with numbers denoting either stimulus positions from left to right, or the colors red, blue, green, and yellow, respectively) was repeated 9 times. Short 30-s breaks were provided in between blocks.

After this training phase all participants were tested in a fully within-subject design for transfer to each of the three cue conditions, that is, a transfer test with just position cues, a transfer test with just color cues, and a transfer test with combined position and color cues. The order of these three transfer tests was varied between participants and taken into account during analyses (the counterbalance procedure was not perfect due to the number of participants). For each transfer test, three blocks of stimuli were presented: a pseudo-random block, a sequence block, and another pseudo-random block. The sequence block in every transfer test involved 4 repetitions of the same 12-item sequence as practiced in the training phase, for a total of 48 trials (less trials were used than in the training phase to reduce sequence learning in the transfer phase). The pseudo-random blocks in each transfer test now consisted of a series of four randomly picked SOC sequences, with no element and sequence repetitions allowed. Again, pseudo-random blocks were never repeated for the same participant. In all other aspects the transfer phase was identical to the training phase.

Finally, participants performed the PDP task (see Destrebecqz & Cleeremans, 2001) with the same response cues as in their training phase but now presented after each keypress as response effects. The PDP consisted of two free generation tasks of 96 key presses, first under inclusion instructions (i.e., participants were required to reproduce as much of the experimental sequence as possible), and subsequently under exclusion instructions (i.e., participants were required to avoid the experimental sequence as much as possible). In the latter task, participants were instructed to refrain from any strategy that might facilitate their task (such as constantly repeating a small and unfamiliar set of key presses). From the notion that awareness can be characterized by control, explicit learning is assumed to be expressed by the difference between inclusion and exclusion performance, while implicit learning should express itself in greater-than-chance sequence reproduction on the exclusion task (Destrebecqz & Cleermans, 2001).

### Results

For each participant and each block, erroneous key presses and correct responses with RTs three standard deviations above the mean RT of the block were excluded from further analyses. This initial procedure eliminated less than 5% of the data in both the acquisition and the transfer phases. Subsequently, for all participants, mean RTs and error percentages (PEs) were calculated for each block in both the training and transfer phases on the basis of the remaining data. Additionally, awareness scores were calculated for both the PDP inclusion and exclusion tasks by counting the number of correctly produced three-element chunks (which constitute the basis of an SOC sequence) and dividing this number by the maximum number of correctly produced chunks of three (which is 94), in order to create an awareness index ranging from zero to one.

#### Awareness

A mixed-design ANOVA on awareness scores, with Task (2; inclusion vs. exclusion) as within-subject variable, and Group (3; position training, color training, and combined training) as between-subject variable, showed a significant main effect of Task, *F*(1, 50) = 6.5, *p* < .05, η_p_^2^ = .12, indicating more correctly produced three-element chunks in the inclusion (mean awareness score = 0.42) than the exclusion task (mean awareness score = 0.38). The Task × Group interaction was not significant (*p* = .44), showing that PDP awareness scores did not reliably differ between the three different training groups. In addition, when each group was divided into the 50% most and 50% least aware participants, the Task × Group interaction was not significant for both more (*p* = .85) and less (*p* = .30) aware participants. Collapsed across the different training groups (as there were no significant group differences), both inclusion, *t*(52) = 5.7, *p* < .001, and exclusion scores, *t*(52) = 4.8, *p* < .001, exceeded chance level (.33; because no repetitions were allowed, only three options remained after each key press). Thus, overall, there are indications of both explicit (i.e., the inclusion score exceeding the exclusion score) and implicit (both inclusion and exclusion scores exceeding chance level) sequence learning, while groups did not differ significantly on awareness scores. Finally, including awareness as a covariate did not affect the analyses reported below. For the sake of brevity, then, it was chosen to not further report on awareness.

#### Training

##### Blocks 2 to 8

**Figure 1.**
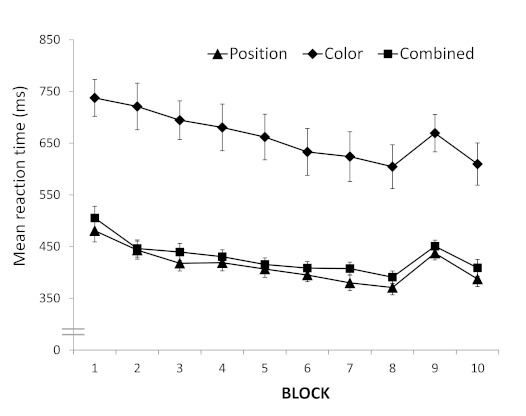
Mean reaction times (in milliseconds) for the position, color, and combined training groups in the training phase of Experiment 1. Error bars depict standard errors.

Mean RTs were analyzed for Blocks 2 to 8 (see Figure 1) in a mixed-design ANOVA with Block (7) as within-subject variable and Group (3; position training, color training, and combined training) as between-subjects variable. Main effects were observed for both Block, *F*(6, 300) = 34.8, *p* < .001, η_p_^2^ = .41, and Group, *F*(2, 50) = 31.2, *p* < .001, η_p_^2^ = .56. The effect of Block confirms learning during training. A trend towards significance was observed between Block and Group (*p* = .07), suggesting more improvement for the color training group than the other two training groups. This may be explained by taking into consideration the arbitrary color to response mapping, the learning of which accelerated responses more with practice than the highly compatible position to response mapping. With regard to the Group main effect, separate ANOVAs revealed that the color training group responded more slowly than either the position training group, *F*(1, 32) = 35.6, *p* < .001, η_p_^2^ = .53, or the combined training group, *F*(1, 33) = 33.9, *p* < .001, η_p_^2^ = .51. No difference was observed between the position training group and the combined training group (*p* = .44). Similar analyses on PEs did not reveal any significant effects; across the three different training groups PEs never exceeded 4%.

##### Blocks 8 to 10

The critical comparison with respect to sequence learning is the difference between the mean of Block 8 and 10, and Block 9 (see Figure 1; position training group: 58 ms; color training group: 61 ms; combined training group: 51 ms). A mixed-design ANOVA was performed with Block (2; mean of Block 8 and 10, vs. Block 9) as within-subject variable and Group (3; position training, color training, and combined training) as between-subjects variable. Significant main effects were found for Block, *F*(1, 50) = 114.0, *p* < .001, η_p_^2^ = .70, and for Group, *F*(2, 50) = 30.4, *p* < .001, η_p_^2^ = .55. The main effect of Block indicates sequence learning, whereas the main effect of Group was rooted in reliably slower responding in general for the color training group than either the position training, *F*(1, 32) = 35.8, *p* < .001, η_p_^2^ = .53, or the combined training groups, *F*(1, 33) = 32.7, *p* < .001,η_p_^2^ = .50. Importantly, the Block by Group interaction was far from significant (*p* = .67), suggesting that sequence learning was not reliably different between training groups (a similar analysis on normalized scores also did not produce a significant Block by Group interaction; see Robertson & Pascual-Leone, 2001). As noted above, however, the crucial analyses for exploring differences in sequence learning between training groups are related to the transfer scores below.

A similar mixed-design ANOVA on PEs resulted in a significant Block main effect, *F*(1, 50) = 11.7, *p* < .01, η_p_^2^ = .19, indicating less errors for the sequence blocks. Again, sequence learning did not reliably differ between training groups (*p* = .70), with PEs never exceeding 4%.

#### Transfer

**Figure 2.**
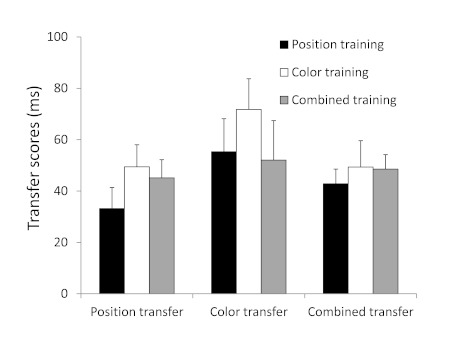
Mean transfer scores (in milliseconds) for the different training groups across transfer tests in Experiment 1. Transfer scores reflect the difference in performance between sequentially and (pseudo-)randomly structured blocks of trials within the transfer phase. Error bars depict standard errors.

**Figure 3.**
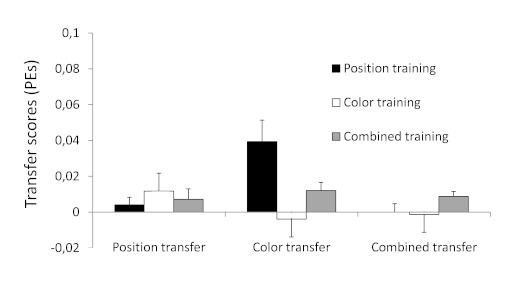
Mean transfer scores (error percentages, PEs) for the different training groups across transfer tests in Experiment 1. Transfer scores reflect the difference in performance between sequentially and (pseudo-)randomly structured blocks of trials within the transfer phase. Error bars depict standard errors.

Transfer scores (see Figures 2 and 3) were calculated for each participant and each transfer test (i.e., position transfer, color transfer, and combined transfer) by taking the difference in RT and PE between the sequence block and its two surrounding pseudo-random blocks. The order of performing the three transfer tests had no influence on transfer scores (*ps* ≥ .20), and is not included in the report of the subsequent analyses. First, we performed one-sample *t*tests (test-value = 0) on all transfer scores, separately for each group, to determine if significant transfer occurred. This showed significant and positive transfer to all three cue conditions for the position training group, *ts*(17) > 4.3, *ps* < .01, the color training group, *ts*(15) > 4.8, *ps* < .001, and the combined training group, *ts*(18) > 3.4, *ps* < .01. The same analyses on PEs (which never exceeded 5% on average across blocks and training conditions) showed significant positive transfer for the position training group on the color transfer test, *t*(17) = 3.3, *p* < .01, for the color training group on the position transfer test, *t*(15) = 2.9, *p* < .05, and for the combined training group on both the color transfer test, *t*(18) = 2.7, *p* < .05, and the combined transfer test, *t*(18) = 3.1, *p* < .01.

In order to answer our major research question whether redundant response cues enhance sequence learning relative to single response cues, we explored performance of the three training groups measured on identical tasks. Separate MANOVAs for RTs and PEs were performed with the three transfer scores (position transfer, color transfer, and combined transfer) as multiple dependent measures, and with Group (3; position training group, color training group, and combined training group) as a fixed factor. MANOVA was used because we are interested in comparing the three training groups across three different test moments in order to obtain a clear indication of relative sequence learning effects, and MANOVA allows for more sensitive testing by considering all effects in one analysis. The effect of Group was not significant for transfer scores on RTs (*p* = .78; separate univariate tests all demonstrated *ps* .30), but there was a significant effect of Group for the transfer scores on PEs, *F*(6, 98) = 3.0, *p* < .01, η_p_^2^ = .16, that was rooted in a significant Group effect on the PEs of the color transfer test (which becomes immediately clear from Figure 3), *F*(2, 50) = 6.9, *p* < .01, η_p_^2^ = .22, but not on the position or combined transfer test (*ps* > .30). Specifically, this significant Group effect on the color transfer test for PEs originated from significant differences in transfer between the position training group (mean transfer score = 0.039) and the color training group (mean transfer score = -0.004), *t*(32) = 3.1, *p* <.01; between the position training group and the combined training group (mean transfer score = 0.012), *t*(35) = 2.2, *p* < .05; and between the color training group and the combined training group, *t*(33) = 2.1, *p* < .05. Please note, however, that the transfer effect on PEs of the color training group to the color transfer test may have been affected by the relatively large (though not significantly so; see Figure 2) transfer on RTs, possibly indicating some trade-off between transfer on RTs and PEs.

Thus, overall, transfer was similar for the three training groups on all transfer tests for RTs, and almost all the transfer tests for PEs. This strengthens the findings from the training phase that sequence learning was not modulated by cue condition during training.

### Discussion

In Experiment 1 we aimed at exploring the effect of redundant response cues on sequence learning in an SRT task. Specifically, we employed a training condition in which both the position and the color of the stimulus signaled the correct response, and compared sequence performance to that under single response cue (i.e., position or color) training conditions. Subsequently, for all participants we assessed the transfer of sequence knowledge to all three response cue conditions. The main result of Experiment 1 is that we did not observe any indication that sequence learning benefited from training with combined position and color response cues (in fact, learning was numerically smallest in this group) as compared to learning with single position or color response cues. Furthermore, there was no indication that the different training conditions produced different levels of sequence awareness. In the training phase, participants training with either position, color, or combined response cues all showed comparable amounts of sequence learning on both RT and accuracy measures. The transfer tests strengthened this notion as sequence learning was still highly comparable between training groups when performing the task under identical response cue conditions, with the only exception to this being the accuracy measure on the color transfer test. The latter finding deserves some further elaboration.

In terms of accuracy it was observed that the position training group showed a better transfer to color response cues than the combined training group, whereas transfer of both was better than that of the color training group. This probably does not reflect a difference in the amount of sequence learning between groups, as across all other transfer tests (RTs and PEs) there were no significant differences. We believe these differences in transfer rather reflect the amount of experience with the color response cues and their arbitrary mapping to responses. Obviously, the color training group already acquired the arbitrary mapping between colors and responses before entering the color transfer test, and could perform this transfer test without much effort (i.e., producing few errors). On the other hand, the position training group had no experience whatsoever with the color to response mapping during the training phase. Moreover, whereas this group could use their sequence knowledge in the sequence block of the transfer test to (partly) circumvent this mapping, they had to fully rely on this mapping during the random blocks of the transfer test. This can possibly explain the relatively large difference between sequence and random blocks on PEs for the position training group. Most interestingly, from this reasoning it seems that the participants from the combined training group gained some benefit from their exposure to the color response cues during their training session, in that they learned the mapping between colors and responses already to some extent. Thus, it seems that even though the color response cues were not facilitating baseline response latencies or sequence learning (possibly because the arbitrarily mapped color response cues were not selected for action as position cues are more stimulus-response compatible), the color response cues were not completely ignored either in the combined training condition.

The findings of Experiment 1 are in line with those of Abrahamse et al. (2009) in that congruent and temporally synchronized response cues do not facilitate sequence learning. Abrahamse et al. (2009) employed redundant visual and tactile cues, with the latter being presented directly to the fingers. Whereas the absence of better sequence learning with redundant cues in that study may have been explained by the spatial disparity of both cues (thereby preventing successful integration), the current study employed temporally synchronized cues that were presented at the same location (i.e., the color and the position of the stimulus) and still sequence learning was unaffected by cue redundancy. This is further support for the claim that sequence learning in the SRT task does not benefit from redundant sensory cues.

However, the results of Experiment 1 can still be explained by differences between response cues in baseline response latencies. If, in line with Keele et al. (2003), multiple (stimulus- and response-based) learning systems are involved in sequence learning in the SRT task - and sequence learning effects arise independently in each of these - the absence of any observed benefit from redundant response cues on general performance can be explained by assuming that one of the (in this case stimulus-based) systems is too slow to contribute to general performance. Indeed, in both the study of Abrahamse et al. (2009) and in Experiment 1 of the current study, one of the single cue conditions (i.e., tactile and color cues, respectively) produced much slower responses on average than the other single cue condition (i.e., visual position cues). Such a “race” account could explain why redundant cue conditions did not affect sequence performance as compared to single cue conditions in the study by Abrahamse et al. (2009) and Experiment 1 of the current article.

Experiment 2 aimed to further explore the issue of redundant sensory information in the SRT task by employing response cues that produced more or less comparable baseline latencies. If an overall absence of learning benefits by using redundant response cues underlies the results from Abrahamse et al. (2009) and Experiment 1 of the current study, then we would expect to also find no learning benefits in Experiment 2. However, if the above mentioned race account interfered with the expression of such benefits, then using two response cues with similar baseline RT should surface these learning benefits.

## EXPERIMENT 2

In Experiment 2, we opted for using shape and color features of stimuli, as these are both arbitrarily mapped onto responses and thus were expected to produce more or less comparable baseline response latencies. Indeed, in a small within-subject pilot study on random sequences of stimuli, the shape and color features provided highly similar baseline RTs. If there exist different stimulus-based (in this case for both shape and color) learning systems in which sequence-specific processing gains develop with practice, than it would be predicted that the sequence learning effect is larger for the condition with combined shape and color response cues than for either single response cue conditions. In addition to this change of response cues, Experiment 2 also employed a different transfer phase than Experiment 1. In Experiment 1we assessed transfer across all response cue conditions, and the motivation for this was to provide a significant comparison of sequence learning between different training groups while circumventing the problem of different baseline response latencies. However, with the pair of response cues in Experiment 2 this was no longer necessary (i.e., providing comparable baseline response latencies during the training phase was the whole purpose of Experiment 2). In Experiment 2 we assessed transfer to a cue condition that was new for all participants, namely responding to position response cues, in order to determine if purely response-related learning developed, and in order to compare the amount of purely response-related learning across the different training groups. The rationale is that testing in a new response cue condition would allow transfer only of purely response-related learning (e.g., response location learning) and not of sequence learning that is specific to the response cues from the training phase (e.g., shape- or color-related sequence learning). This transfer method to explore the nature of sequence learning has been used before in various studies (e.g., Abrahamse et al., 2008; Cohen, Ivry, & Keele, 1990; Keele, Jennings, Jones, Caulton, & Cohen, 1995; Willingham, 1999; Willingham et al., 2000).

In sum, the main purpose of Experiment 2 was to explore the potential sequence learning benefit from congruent shape and color response cues as compared to sequence learning in single response cue conditions (i.e., either shape or color). This enabled to test the hypothesis that the absence of redundancy benefits in Experiment 1 (as well as in the study by Abrahamse et al., 2009) was related to different baseline RTs per response cue - in which case we would expect to find redundancy benefits in Experiment 2. In addition, a second purpose of Experiment 2 was to provide additional support for a purely response-related component of sequence learning, and to explore whether this response-related component contributed equally to overall learning across all training groups. Apart from the change in response cues and the transfer design, Experiment 2 was very similar to Experiment 1.

### Method

#### Participants

Sixty undergraduates (52 women, eight men; *M*_age_ = 23, *SD* = 3.3; three left-handed) from the University of Finance and Management (Warsaw, Poland) gave their informed consent to participate in the experiment in exchange for course credits. They had normal or corrected to normal visual acuity, scored perfectly on a subset of the Ishihara color blindness test (Ishihara, 1993), and were naďve as to the purpose of the study.

#### Stimuli and apparatus

In Experiment 2, the single placeholder consisted of a single grey-lined square (3 × 3 cm) filled with white presented on a black background. In the shape condition, stimuli consisted of the appearance in the square placeholder of a purple colored circle, diamond, cross, or triangle (sized all to just fit the placeholder). In the color condition, stimuli consisted of the appearance in the square placeholder of a circle that was colored either red, blue, green, or yellow. Finally, in the combined condition, stimuli consisted of the appearance in the square placeholder of a green diamond, a red cross, a yellow circle, or a blue triangle. There were no further differences with Experiment 1.

#### Procedure

The procedure of Experiment was highly similar to that of Experiment 1, with differences concerning only the stimulus conditions in the training phase, and the design of the transfer phase. Only these differences will be reported here.

Participants were randomly assigned to one of three experimental groups for the training phase, in which an SRT task was performed: the shape training group (*n* = 20), the color training group (*n* = 20), or the combined training group (*n* = 20). In the shape training group, participants were instructed to respond to the shape of a purple colored stimulus, with a diamond, a cross, a circle, and a triangle corresponding to the [*c*], [*v*], [*b*], and [*n*] keys of a standard QWERTY keyboard, respectively. In the color training group, participants were instructed to respond to the color of a circle, with the colors green, red, yellow, and blue corresponding to the [*c*], [*v*], [*b*], and [*n*] keys, respectively. In the combined training group, each of the four shapes (i.e., diamond, cross, circle, triangle) was presented in a unique color (i.e., green, red, yellow, blue), so that participants could respond to either the shape or to the color of the stimulus, or to both.

After the training phase all participants were tested for transfer to a visual SRT task with spatial stimuli. In this transfer phase, three blocks of stimuli were presented: a pseudo-random block, a sequence block, and another pseudo-random block. The sequence block involved 4 repetitions of the same 12-item sequence as practiced in the training phase, for a total of 48 trials (less trials were used than in the training phase to reduce sequence learning in the transfer phase as much as possible). The pseudo-random blocks in each transfer test again consisted of a series of four randomly picked SOC sequences, with no element and sequence repetitions allowed. These pseudo-random blocks were never repeated for the same participant. In all other aspects the transfer phase was identical to the training phase. Finally, participants performed the PDP task with the same response cues (now as response effects) as in their training phase.

### Results

The initial exclusion procedure (see Experiment 1) eliminated less than 5% of the data in both the acquisition and the test phases. Mean RTs and PEs were calculated for all participants for each block in both the training and transfer phases on the basis of the remaining data. Awareness scores were calculated as in Experiment 1.

Four participants were excluded from the analyses reported below. Two participants from the color training group had great difficulty performing the SRT task with the arbitrary color stimuli; for one participant this resulted in average block RTs all above 1,200 ms, whereas for the other participant this resulted in never reaching ave-rage block PEs lower than ten percent. Two participants of the shape training group showed near perfect sequence awareness in the PDP task: They reached perfect reproduction of the sequence in the inclusion task, and succeeded well in avoiding reproduction in the exclusion task. Even though it is inevitable that some sequence awareness develops in an SRT task with deterministic sequences, these participants were clear outliers. Because explicit knowledge has been found to behave qualitatively different than implicit knowledge (e.g., Jiménez, Vaquero, & Lupiáńez, 2006), we decided to exclude these to keep the different training groups comparable with respect to awareness. Hence, only 18 participants remained for the shape and color training groups.

#### Awareness

A mixed-design ANOVA on awareness scores, with Task (2; inclusion vs. exclusion) as within-subject variable, and Group (3; shape training group, color training group, and combined training group) as between-subject variable, produced a significant Task main effect, *F*(1, 53) = 72.2, *p* < .001, η_p_^2^ = .58; inclusion scores (mean awareness score = 0.50) exceeded exclusion scores (mean awareness score = 0.36). An absent Task × Group interaction (*p* = .38) showed that awareness scores did not reliably differ between training groups. In addition, when each group was split half based on awareness scores (see Experiment 1), the Task × Group interaction was not significant for both more (*p* = .20) and less (*p* = .70) aware participants. Collapsed across groups, both inclusion, *t*(55) = 10.6, *p* < .001, and exclusion scores, *t*(55) = 2.5, *p* < .05, exceeded chance level (.33). Thus, overall, there seem to be indications of both explicit (i.e., the inclusion score exceeding the exclusion score) and implicit (both inclusion and exclusion scores exceeding chance level) sequence learning, while groups did not differ significantly on awareness scores. As before, including awareness as a covariate did not affect the analyses reported below, and it was chosen to not further report on awareness.

#### Training

##### Blocks 2 to 8

**Figure 4.**
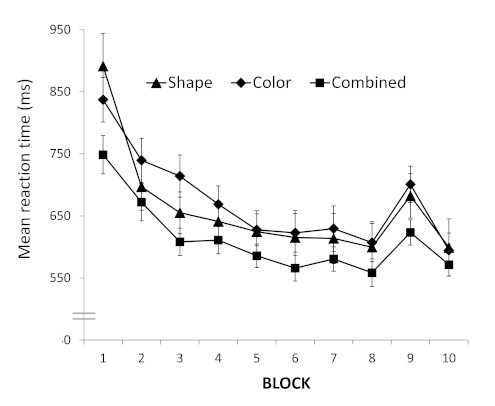
Mean reaction times (in milliseconds) for the position, color, and combined training groups in the training phase of Experiment 2. Error bars depict standard errors.

Mean RTs were analyzed for Blocks 2 to 8 (see Figure 4) in a mixed-design ANOVA with Block (7) as within-subject variable and Group (3; shape training group, color training group, and combined training group) as between-subjects variable. This indicated only a significant main effect for Block, *F*(6, 318) = 29.7, *p* < .001, η_p_^2^ = .36. The Group main effect and the Block ×Group interaction effect were not significant (ps .30). Hence, this shows that we succeeded in comparing different cue conditions with more or less similar baseline RTs. A similar ANOVA on PEs did not produce significant results (PEs never exceeded 3%).

##### Blocks 8 to 10

To answer the major research question of Experiment 2 whether there are differences in sequence learning effects between the different training groups (see Figure 4; shape training group: 83 ms; color training group: 100 ms; combined training group: 60 ms), we performed a mixed-design ANOVA with Block (2; mean of Block 8 and 10, versus Block 9) as within-subject variable and Group (3; shape training group, color training group, and combined training group) as between-subject variable. A significant effect was found only for Block, *F*(1, 53) = 42.9, *p* < .001, η_p_^2^ = .45, indicative of sequence learning. The Group main effect as well as the Block by Group interaction effect were not significant (*ps* ≥ .30). The latter findings indicate more or less similar sequence learning effects across the different training groups. A similar mixed ANOVA on PEs produced no significant results, but across the three different training groups PEs for Blocks 8 to 10 never exceeded 3%.

#### Transfer

**Figure 5.**
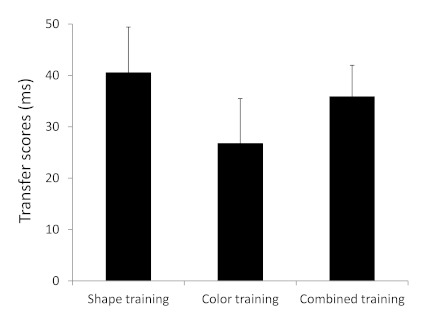
Mean transfer scores (reaction time, RTRT) across the different training groups of Experiment 2. Transfer scores reflect the difference in performance between sequentially and (pseudo-)randomly structured blocks of trials within the transfer phase. Error bars depict standard errors.

For each participant we calculated a transfer score (see Figure 5) by taking the difference in RT and PE between the sequence block and its two surrounding pseudo-random blocks from the transfer phase. One-sample *t*tests (test-value = 0) were performed for each training group, to determine whether significant transfer had occurred. This showed positive transfer for the shape training group (transfer score = 41 ms), *t*(17) = 4.6, *p* < .001, for the color training group (transfer score = 27 ms),*t*(17) = 3.1, *p* < .01, and for the combined training group (transfer score = 36 ms), *t*(19) = 5.8, *p* < .001. The same analyses on PEs did not show significant results (*ps* ≥ .15), but transfer was always positive in absolute terms, and PEs never exceeded 3% across the transfer blocks. Most importantly, the amount of transfer (both for RT and PE) was not significantly different between the training groups (*ps* ≥ .45).

### Discussion

Experiment 2 aimed at exploring the possibility that the use of redundant response cues results in independent sequence learning effects across multiple stimulus-based learning systems (in addition to response-based learning), but that this effect might have been obscured in previous experiments because one of the stimulus-based systems was too slow to contribute to general performance. This notion arose from the study of Abrahamse et al. (2009) and Experiment 1 of the current paper, both in which one response cue was responded to much slower on average than the other. Therefore, in Experiment 2 we chose response cues that - when employed separately - provide more or less similar baseline response latencies. The main conclusion from Experiment 2 is that redundant shape and color response cues, though indeed providing very similar baseline response latencies, do not enhance sequence learning in the SRT task as compared to single shape or color response cues. This runs counter to the type of race account described above, and further supports the claim that sequence learning in the SRT task does not typically benefit from redundant sensory information.

An additional finding of Experiment 2 is that transfer occurred from the different training conditions (i.e., shape, color, or combined) to a test with new response cues (i.e., visual position response cues), but the amount of transfer did not reliably differ between training groups. This indicates that a response-related component of sequence learning developed (cf. Willingham, 1999; Willingham et al., 2000) - possibly in addition to more stimulus-based components - and that this component was of similar size across the three training groups.

## GENERAL DISCUSSION

The role of sensory information in sequence learning is one of the major issues of debate within the SRT literature (Abrahamse et al., 2010; Clegg et al., 1998). The current paper contributed to this issue by exploring potential sequence learning benefits from the availability of redundant sensory information. From the notion that sensory information plays a significant role in sequence learning (it has been shown that sequence learning can be based on sensory information; e.g., Clegg, 2005; Remillard, 2003), it may be predicted that sequence learning can benefit from the availability of redundant sensory information, at least under some conditions. However, in a study by Abrahamse et al. (2009) no sequence learning benefits were observed from adding congruent (i.e., redundant) tactile response cues to an otherwise standard SRT task with visual position response cues. Abrahamse et al. (2009) acknowledged that, on the base of their results, the strong claim that sequence learning is typically unaffected by redundant sensory information would be premature. The current study aimed at further exploring this issue in two experiments. Experiment 1 showed that no additional sequence learning benefits are observed when redundant position and color response cues are presented at the same location. Experiment 2 additionally showed that response cues with similar baseline response latencies also leave the magnitude of sequence learning unaffected. From the set of experiments that are reported here and in the study of Abrahamse et al. (2009), then, we believe that there is by now substantial justification for the claim that sequence learning in the SRT task does not benefit from redundant sensory information, at least at the current level of practice. We will now discuss our findings in relation to the representational nature of sequence learning, both with respect to the informational content that underlies sequence representations, and with respect to the implicit-explicit division.

### Stimulus- and response-based learning

In the SRT literature, ample empirical evidence exists for both stimulus- and response-based learning (Abrahamse et al., 2010). The current study, however, provides little indications for stimulus-based learning. First, as noted above, no learning benefits were observed when redundant sensory information was available. Second, various instances of positive transfer were observed between different stimulus settings. Across the literature, transfer testing is probably the major tool in determining the level at which sequence learning occurs - the rational being that transfer only occurs when the major level(s) of learning are not affected between practice and transfer. These transfer results thus indicate that learning was predominantly response-based (e.g., based on response locations or response-effects), and that stimulus-based learning barely developed. The latter conclusion clearly discredits the claim that implicit learning is a fully unselective process(e.g., Keele et al., 2003; Reber, 1993), and pushes future research to answer the question about what is determining the relative weights of the multiple potential response and stimulus features as the building blocks of sequence representations across different studies.

An interesting option in this respect is the suggestion that implicit learning may be restricted to active features of task processing - that is, to the features of the task set. Task set consists of those representations that are actively maintained during task execution, comprising both the overall goal of a task and the more detailed characteristics such as relevant stimulus and response features and stimulus-response mappings (see Monsell, 2003; Sakai, 2008). Abrahamse et al. (2010) reviewed a large literature and concluded that sequence learning probably is not limited to a single type, as ample empirical support exists for multiple types of learning (e.g., response location learning, response-effect learning, stimulus-based learning). They proposed that implicit learning should be understood as an associative process that is directed by top-down selection of feature (both stimulus and response) maps - building from the point of view that the brain processes information in a distributed manner (Hommel, Müsseler, Aschersleben, & Prinz, 2001). Hence, implicit learning is restricted to feature maps that are (most) relevant for the current task, thereby providing strong selectivity.

An explanation in terms of task set is closely related to the issue of selective attention. Indeed, if one assumes that the particular task set drives selective attention processes (cf. intentional weighting; see Hommel et al., 2001), it may be argued that stimulus-based learning is contingent upon attentional selection. Such a relationship between selective attention and implicit learning has been shown by Jiménez and Méndez (1999), who claimed that stimulus features need to be attentionally selected to become associated. Specifically, Jiménez and Méndez (1999) employed a design in which on each trial one of four different shapes was presented at one of four locations. Participants were responding to a sequence of stimulus locations, but, in addition, there was a contingency between the shape of the stimulus and the next stimulus location. It was observed that the latter contingency was learned only when the shape-feature was made task-relevant by a secondary counting task. Possibly, in the current study attention was mostly directed to response locations (e.g., because response generation took up most of the available attentional resources; cf. Deroost & Soetens, 2006a), thereby avoiding any benefits from (redundant) response cues. This notion would indicate that a pair of redundant response cues only enhances sequence learning when both are attentionally selected, that is, when they are both an integral part of the task set.

A substantial role for task set in implicit learning processes thus seems to be a notion that is worthwhile considering and in need of further exploration. Moreover, an explanation of implicit learning in terms of associative learning that is restricted to the most active features, would safeguard us from Mackintosh’ (1978) fear that simple associations would put us “at the mercy of … chance conjunction between events” (p. 54), and therefore would provide functional selectivity to an otherwise automatic, associative process.

Alternatively, one could explain current results by arguing that stimulus-based learning is (predominantly) restricted to spatial information. For example, it could be that stimulus-based learning is solely due to anticipations in the shifting of attention to relevant locations. Indeed, most studies that provided support for stimulus-based sequence learning employed spatial stimuli. Moreover, Koch and Hoffmann (2000) found a clear advantage for learning of spatial sequences (either stimulus-based or response-based) over symbolic sequences. However, there are some studies that show (response-independent) sequence learning with non-spatial stimuli (e.g., Frensch & Miner, 1995; Goschke & Bolte, 2007), whereas Koch and Hoffmann (2000) actually also observed small but significant learning with symbolic stimuli. Moreover, it would be difficult to see why response-effect learning in the SRT task (e.g., Ziessler & Nattkemper, 2001) would be restricted to spatial response-effects. Finally, Keele et al. (2003) did not restrict their model to spatial information per se. All of this clearly requires further exploration.

### Implicit versus explicit learning

Another major topic of debate in the SRT literature concerns the dichotomy of implicit and explicit sequence learning. On the one hand, the debate has focused on the methodological issue of empirically disentangling implicit and explicit contributions to performance. Though the field is still far from reaching consensus, we here argued that the PDP is the most sophisticated tool to date for this purpose, as it considers the lack of process purity of tasks (i.e., most tasks involve both implicit and explicit processes; Destrebecqz & Cleeremans, 2001). On the other hand, it is extensively debated as to whether implicit and explicit learning are qualitatively different from each other (e.g., Jiménez et al., 2006). Though it is not the primary issue of the current paper, we here briefly discuss our findings on awareness in this respect.

In both experiments reported here we observed a mix of implicit and explicit learning: Participants produced on average more correct fragments of the sequence under inclusion than under exclusion instructions, but were nevertheless not able to fully prevent such production under exclusion instructions. Importantly, however, awareness differences appeared to have no impact on the various response cue manipulations that we introduced across Experiments 1 and 2 (in particular, awareness as a co-variate did not produce interactions). Though we need to be conservative with respect to such null findings (especially because we worked with a relatively small sample of participants for the purpose of testing the role of awareness, possibly with larger power results would have been different), it may be tentatively concluded that implicit and explicit learning processes behaved very similar in the current study as (a) neither implicit nor explicit learning was enhanced by providing redundancy in response cues, and (b) both implicit and explicit learning transferred across the various stimulus conditions that we presented. With respect to the former conclusion, the absence of such redundancy benefit is surprising to the extent that implicit learning is not typically understood as being very selective (Keele et al., 2003; Reber, 1993). Moreover, it seems that response cue redundancy did not increase the saliency of regularity, as explicit learning was also unaffected by it (indeed, it could even be argued that possibly explicit learning was less pronounced in the combined training groups because larger stimulus variation impaired conscious hypothesis testing; cf. Norman, Price, Duff, & Mentzoni, 2007).

In conclusion, the current study provides further support for the notion that implicit sequence learning in the SRT task is not typically enhanced when presenting redundant response cues. In combination with the study by Abrahamse et al. (2009), this absence of a redundancy benefit has been observed for pairs of tactile/(visual-)position, position/color, and color/shape response cues. These observations are not easily reconcilable with the growing consensus that stimulus-based sequence learning plays an important role in the SRT task. A potential explanation holds that implicit sequence learning is strongly affected by (top-down) influences of task set, thereby providing substantial selectivity.

## Footnotes

1 We use the term response cue because in the current study multiple features of a single stimulus simultaneously signal the correct response.
